# Patient-specific 3D-tissue slices from peritoneal metastases – An *in vitro* model for individual susceptibility analysis

**DOI:** 10.1515/pp-2024-0012

**Published:** 2025-02-26

**Authors:** Christian Etzold, Orestis Lyros, Matthias Mehdorn, Robert Nowotny, Stefan Niebisch, Boris Jansen-Winkeln, Katrin Schierle, Ines Gockel, René Thieme

**Affiliations:** Department of Visceral, Transplant, Thoracic and Vascular Surgery, University Hospital of Leipzig, Leipzig, Germany; Department of General, Visceral and Oncological Surgery, St. Georg Hospital Leipzig, Leipzig, Germany; Institute of Pathology, University Hospital of Leipzig, Leipzig, Germany; Institute of Pathology, SLK-Kliniken Heilbronn, Heilbronn, Germany

**Keywords:** gastric cancer, peritoneal metastases, tissue-slice, chemotherapy response, pressurized intraperitoneal aerosol chemotherapy

## Abstract

**Objectives:**

The prognosis of patients with peritoneal metastases (PM) is poor, and these patients have a brief overall survival. Most patients with advanced PM receive palliative therapy to maintain their quality of life. In our current study, we investigated whether patient-specific 3D-tissue slices from patients with PM subjected to pressurized intraperitoneal aerosol chemotherapy could be cultured *in vitro*.

**Methods:**

Biopsies from gastric cancer patients with PM were characterized for cytokeratin-positive tumor cells and the proliferation marker Ki-67. Biopsies from seven patients were cut to 350 µM thick slices in a standardized manner, cultured with 10 µM 5-fluorouracil, doxorubicin, cisplatin, oxaliplatin, or irinotecan for 96 h, and then examined histopathologically and via immunohistochemistry for persistent cytokeratin and Ki-67 expression.

**Results:**

*In vitro* cultured slices revealed a similar morphology to un-cultured specimens, and Ki-67-positive tumor cell areas were present after 96 h. The total amount of tumor cells per slice was determined by pan-cytokeratin staining. In the doxorubicin-treated slices, the cytokeratin-positive tumor cell fraction and proliferative (Ki-67pos) cells were decreased. Patient-specific 3D-tissue-slice cultures from peritoneal biopsies were cultured *in vitro* for up to 4 days.

**Conclusions:**

Potentially, these cultures are a reliable model to evaluate the chemosensitivity of patients with PM. Further investigation is needed to match the chemosensitivity with the clinical course of these patients.

## Introduction

Worldwide, gastric cancer (GC) is the fifth most frequently diagnosed malignancy and the third leading cause of cancer-associated death [1]. Per year, GC is newly diagnosed in approximately one million patients and causes approximately 748,000 deaths worldwide [1]. GC is related to risk factors, including lifestyle aspects (e.g. smoking, alcohol, and low physical activity), genetic factors (e.g. mutations and polymorphisms), stomach infections and inflammations (e.g. type A and type B [*Helicobacter pylori*-associated] gastritis) and demographics (e.g. socioeconomic status, income, and ethnicity) [2]. The therapeutic options are limited owing to GC’s often late diagnosis at an advanced stage [3]. About 30–40 % of GC patients with stages II to III disease are already first diagnosed with peritoneal metastasis (PM), which must be assessed during clinical staging via diagnostic laparoscopy [4], 5]. However, the frequency of intrapulmonary, intraperitoneal, and intrahepatic metastases varies among age groups: in particular, lung metastases (strong increase > 65 years) and liver metastases (strong increase > 75 years) are detected in older patients, whereas a peritoneal malignancy is already detected in over 60 % of younger patients (<60 years) [6] [7]. There is also evidence that in about 60 % of patients with GC, death is caused by progressive PM [5]. Multimodal therapy regimens including systemic chemotherapy have proven effective in lengthening the survival of patients with GC [8]. The prognosis of GC with synchronous or metachronous PM is poor, and 1-, 3-, and 5-year overall survival (OS) has been reported as only 47 %, 6 % and 6 %, respectively, when a PM was diagnosed macroscopically [9]. The only potential curative approach for GC patients with PM is an oncologic radical D2-(D3)-gastrectomy in conjunction with cytoreductive surgery (CRS). The effect of systemic chemotherapy on PMs is rather weak [10], 11]. The value of hyperthermic intraperitoneal chemotherapy (HIPEC) after CRS is being investigated in ongoing and planned clinical trials. The extent of PM is classified by referring to the peritoneal carcinomatoses index (PCI) according to Sugarbaker [12], 13]. Evidence to improve OS after gastrectomy, CRS and HIPEC depends in principle on two factors: the extent of peritoneal infiltration (assessed by the PCI) and the complete resection of macroscopically visible tumor lesions, determined by the completeness of cytoreduction score (CCS) [14]. A recent palliative treatment option was introduced, namely pressurized intraperitoneal aerosol chemotherapy (PIPAC), in addition to systemic chemotherapy [15]. PIPAC enables restricted disease-limiting therapy in cases of advanced PM or with a palliative intention to maximize patients’ quality of life [12]. The aims of this therapeutic option are to alleviate symptoms, control ascites production, and achieve regression of PM [12]. GC patients with PM are usually treated with a standard combination of doxorubicin [12], 16] and cisplatin [17] for PIPAC.

Tissue-slice-culture-models (TSC) are a suitable method for cultivating various tumor entities, such as gastric and esophagogastric junction cancer [18], 19], glioblastoma [20], colorectal cancer [21], ovarian cancer [22], lung cancer [23], and head and neck squamous cell carcinoma [24]. TSCs have also been found to help in detecting cytotoxic effects on tumor tissue [18]. TSC sections from human gastric and esophagogastric junction cancer have been shown to contain viable tumor cells after 6 days in *in vitro* culture [18]. Moreover, a loss of tumor cells and increased apoptosis has been observed after exposure to the cytostatic agents 5-fluorouracil and cisplatin [18].

The purpose of this study was to detect and investigate this cytotoxic effect by using standard cytostatic combination (doxorubicin and cisplatin) on PM tumor tissue from patients with a primary tumor located in the stomach under *in vitro* conditions in TSCs.

## Materials and methods

### Patients

**Figure 1:**
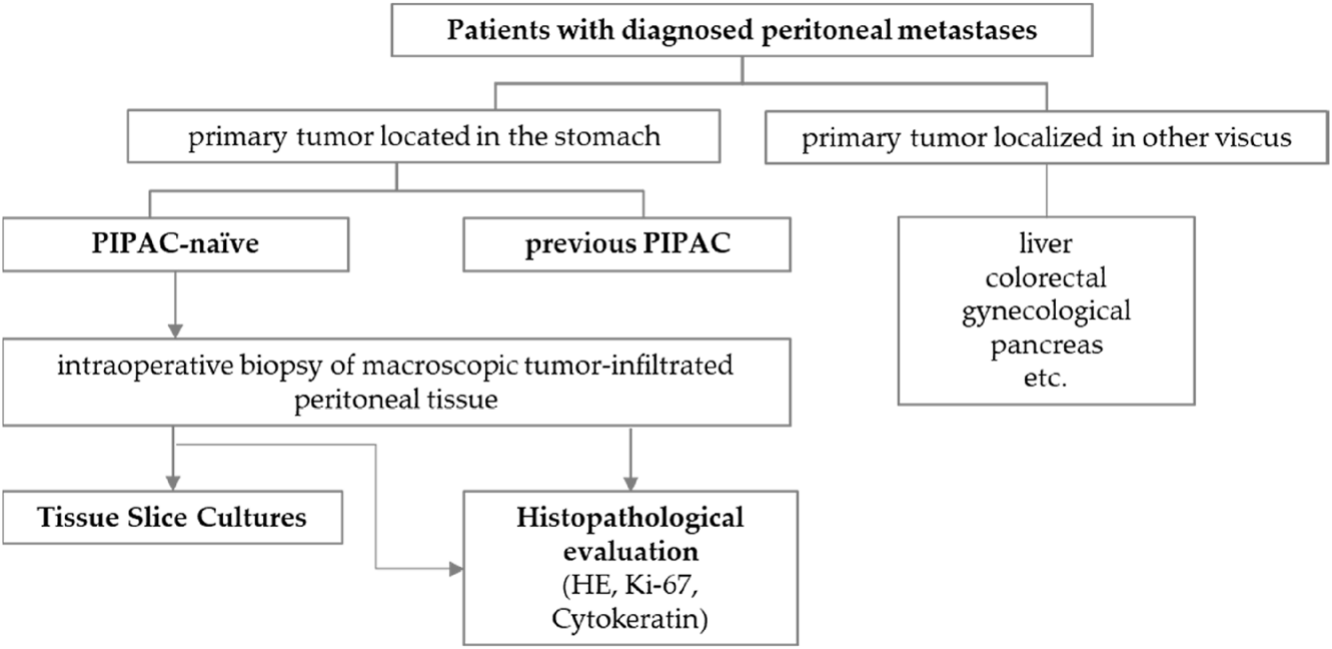
Inclusion criteria and evaluation of GC patients with confirmed PM. Patients with PM were screened for their primary tumor location. Only patients with a primary GC were included in the study. Furthermore, patients were only selected who had not received any PIPAC before (PIPAC-naïve). Biopsies from PM were taken preoperatively, cultured as TSC, and evaluated histopathologically (HE, Ki-67, and for cytokeratin). No TSCs were cultured from patients who already had PIPAC.

We enrolled (n=7) patients with peritoneal metastasized GC in this study (Figure 1). A senior surgeon identified PM lesions during diagnostic laparoscopy, and specimens were collected. Patients’ characteristics are shown in Table 1. All procedures followed were in accordance with the ethical standards of the responsible committees on human experimentation (institutional and national) and with the Helsinki Declaration of 1964 and later versions. The protocol was approved by the local ethics committee of the University of Leipzig, Leipzig, Germany (Approval No. 106-16).

**Table 1:** Patient characteristics.

Variable	Numbers	%
Number of patients	n=7	
Sex		
Male	n=3	(42.9 %)
Female	n=4	(57.1 %)
Age		
Median (range)	53 years	(46–70 years)
Metastases		
PER	n=7	(100 %)
Other than PER		
Liver	n=1	(14.3 %)
Pleura	n=1	(14.3 %)
Ovary	n=1	(14.3 %)
Intestine	n=2	(28.6 %)
Localization of primary tumor^a^		
Cardia	n=3	(42.9 %)
Fundus	n=0	(0.0 %)
Corpus	n=4	(57.1 %)
Antrum	n=1	(14.3 %)
Pylorus	n=0	(0.0 %)
Lauren classification		
Diffuse	n=5	(71.4 %)
Mixed	n=0	(0.0 %)
Intestinal	n=1	(14.3 %)
Not determined	n=1	(14.3 %)
Pre-treatment		
Previous chemotherapy		
2nd lines	n=2	(28.6 %)
1st line	n=7	(100 %)
Gastrectomy	n=5	(71.4 %)
PIPAC procedure		
PCI before 1st PIPAC (median)	26	(2–36)
Ascites before 1st PIPAC		
Median	3,300 mL	(0–5,500 mL)
Average	2,500 mL	(0–5,500 mL)
Pathologic findings in peritoneal biopsies	n=6	(85.7 %)
Percentage of tumor cells in the biopsies		
Minimum	1 %	
Maximum	90 %	
Number of PIPAC procedures		
3 times	n=1	(14.3 %)
2 times	n=5	(71.4 %)
1 time	n=7	(100.0 %)

n, number of patients; c (clinical); TNM – T, tumor, N, node, and M, metastasis category; PER, peritoneum; 1st line, first-line; 2nd line, second-line; PIPAC, pressurized intraperitoneal aerosol chemotherapy; PCI, peritoneal carcinomatosis index (according to Sugarbaker); ^a^ – for one patient two localizations had been determined (cardia and corpus).

### Intraoperative collection of PM specimens

Tissue biopsies for *in vitro* examination from patients with GC and PM were collected together with those for routine histopathological examination during diagnostic laparoscopy, together with a PIPAC procedure [25]. Samples were fixed with 4 % paraformaldehyde (PFA)/PBS at 4 °C for a minimum of 12 h. The tissue samples were embedded in paraffin and trimmed into 5 µm thick slices.

### Preparation of specimens for the tissue slice cultures (TSC) (PIPAC-natïve patients)

**Figure 2:**
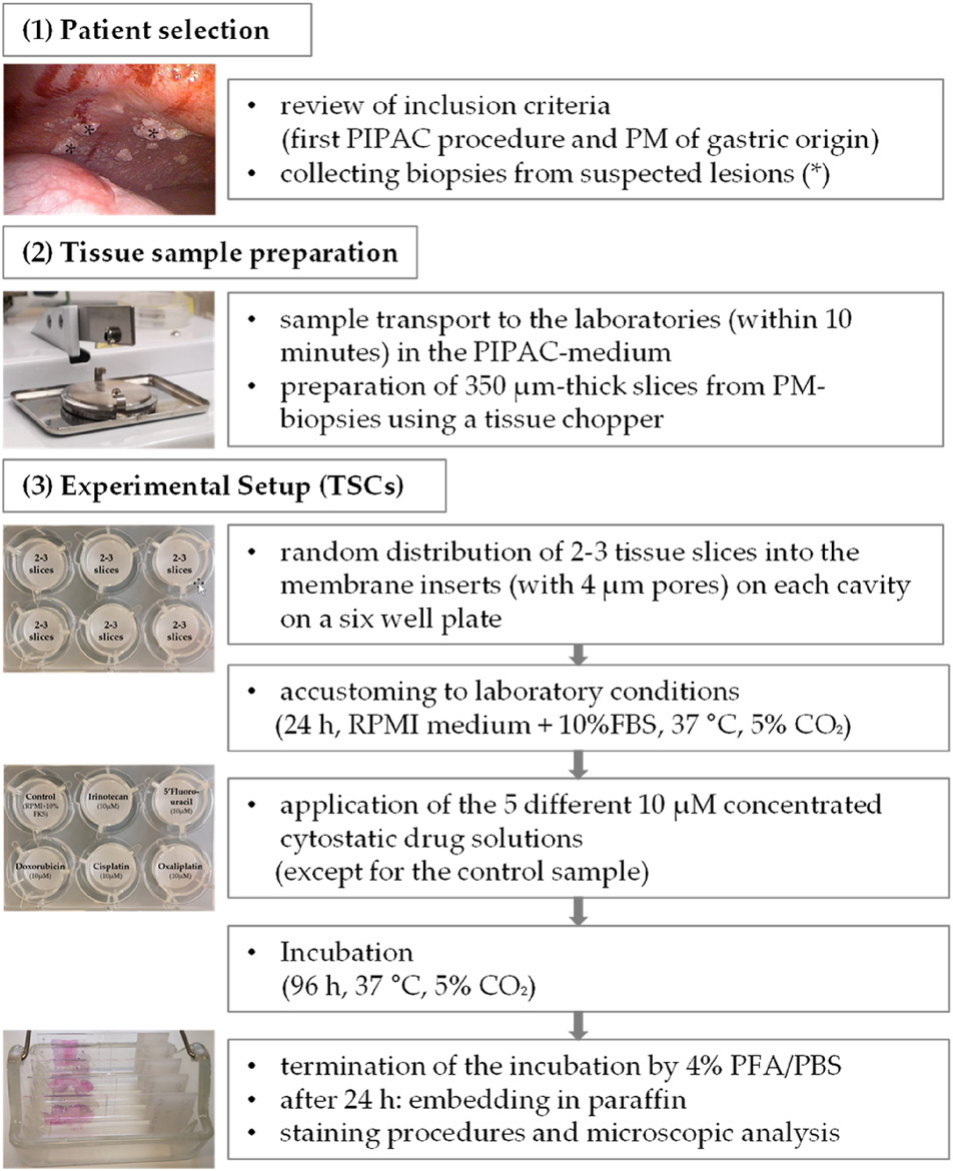
Preparation of TSC and experimental setup. Selection of patients and collection of biopsy samples, preparation of PM-tissue samples and experimental setup (adjustment to the culture conditions, followed by an incubation with cytostatic drugs and histopathological and immunohistochemical examinations).

Specimens for TSC were collected in RPMI/10%FBS/Pen/Strep/Nystatin (ThermoFisher, Darmstadt, Germany) and examined under a stereo-microscope. Non-tumorous tissue was dissected and the specimens cut into 350 µm thick sections by a tissue chopper (McIlwain Tissue Chopper, Cavey Laboratory Engineering, Gomshall, UK). Subsequently, 1 mL of RPMI culture medium was added to each cavity of a 6-well plate containing a membrane insert (pore size: 0.4 µm; GreinerBioOne, Frickenhausen, Germany). Thereafter, 2–3 randomly selected slices were distributed to each well. The slices were cultured at a liquid-air interface, whereby the slices had contact to the medium via the semipermeable membrane, which ensured the supply of nutrients to the tissue. The TSCs were cultured for 24 h (under conditions of 37 °C and 5 % CO_2_) to adapt to the laboratory and cell culture conditions. Afterwards, the TSCs were cultured with 10 µM doxorubicin, cisplatin, irinotecan, oxaliplatin, or 5′-fluorouracil (5-FU) from pharmaceutical preparations (Pharmacy, University Hospital of Leipzig) under standardized conditions of 37° and 5 % CO_2_ for 96 h. All chemotherapeutics were pharmaceutical preparations and prepared freshly (Pharmacy, University Hospital of Leipzig). After treatment, the TSCs were washed with cold PBS, fixed with 4 % PFA/PBS at 4 °C for 12 h, and embedded in paraffin. The embedded samples were trimmed into 5 µm thick slices using a microtome (Figure 2).

### Immunohistochemical staining

The first slice was used for combined Ki-67-protein- and pan-cytokeratin staining. The sections were de-paraffinized (100 % ethanol, 90 % ethanol, 80 % ethanol, 70 % ethanol, water), and endogenous peroxidase was blocked by 3 % H_2_O_2_/MeOH. Afterwards, an epitope retrieval was done by cooking the slices in citrate buffer (10 mM, pH6) for 10 min, followed by blocking with 5 % normal goat serum (Jackson ImmunoResearch, Ely, UK) and a streptavidin/biotin blocking kit (GeneTex, Irvine, US) for 20 min. Primary antibodies against pan-cytokeratin (C2562**,** Sigma-Aldrich, Taufkirchen, Germany, 1:100 in 1 % BSA/TBST) and the Ki-67 protein (HPA000451**,** Sigma-Aldrich, Taufkirchen, Germany, 1:100 in 1 % BSA/TBST) were used (at 4 °C overnight). Slides were rinsed with PBS, and biotinylated secondary antibodies were applied (goat-anti-rabbit, Jackson ImmunoResearch, Ely, UK, 1:1,000 in 1 % BSA/TBST) for 1 h. Slides were washed and incubated with peroxidase conjugated streptavidin (Jackson ImmunoResearch, Ely, UK, 1:1,000 in 1 % BSA/TBST) and alkali phosphatase conjugated goat-anti-mouse (Jackson ImmunoResearch, Ely, UK, 1:500 in 1 % BSA/TBST) for 90 min. After staining, the slides were dehydrated (60 % ethanol, 70 % Ethanol, 80 % ethanol, 90 % ethanol, 100 % ethanol, and xylol) and covered with Mountex (CarlRoth, Karlsruhe, Germany).

### Haematoxylin-eosin (HE) staining

HE staining was performed from the second section of the embedded paraffin block. After deparaffinization and rehydration, the slides were stained with filtrated hematoxylin for 6 min and eosin for 1 min, dehydrated, and covered with Mountex (CarlRoth, Karlsruhe, Germany).

### Analysis of uncultured PM biopsies

HE-stained slices were examined and evaluated under the microscope (Axio Imager A1, Zeiss, Germany). Histopathologic criteria for malignancy were: polymorphia, anisonucleosis, nuclear hyperchromasia, shift in the nuclear-plasma-relation, and increased basophilia. Specimens containing at least one of these criteria were evaluated as tumor-positive (+), otherwise as tumor-negative (−). We also examined the expression levels of Ki-67 and cytokeratin. The slices were grouped, and we determined the proportion of tumor cells to the entire biopsy area (A=0 %, B=1–5%, C=5–50 % and D>50 %). To validate these criteria, PM from 34 patients with GC were stained and characterized.

### Analysis of tissue slice culture samples

Proliferative, active tumor cells were identified via cytokeratin and Ki-67 expression. Moreover, both parameters were compared among the various chemotherapeutic treatment groups. Identifying the tumor cells was accompanied by evaluating the HE-stained slides.

## Results

### Ki-67-protein and cytokeratin expression in biopsies of GC patients with PM

**Figure 3:**
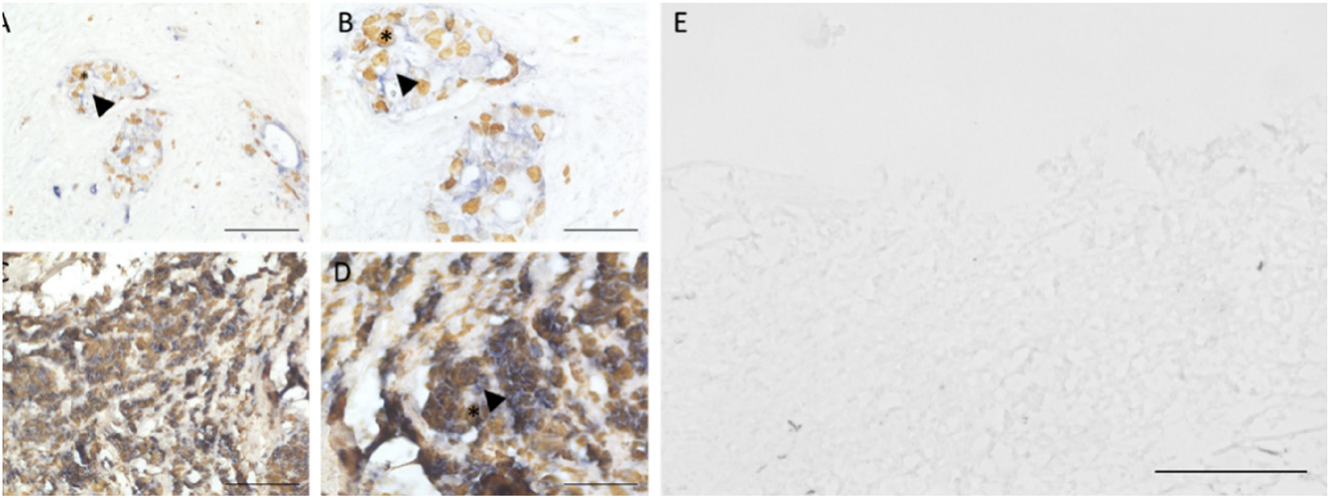
Ki-67 and cytokeratin staining in PM. Histology of a PM biopsies with PM lesions with large quantities of Ki-67 expressing cells (*) and a high expression level of cytokeratin (triangle) (A–D), and non-cancerous peritoneal tissue, which is cytokeratin and Ki-67 negative (E) (bar – 100 µm for A and D, 50 µm for B and D, 500 µm for E).

We assessed the tumor-proliferation marker Ki-67 and epithelial-origin marker (pan-cytokeratin) in PM biopsies. In PM tissue, Ki-67 expressing cells were co-localized in cytokeratin-positive areas (Figure 3A–D). Non-malignant peritoneal tissue was negative for both, cytokeratin and Ki-67 (Figure 3E).

**Figure 4:**
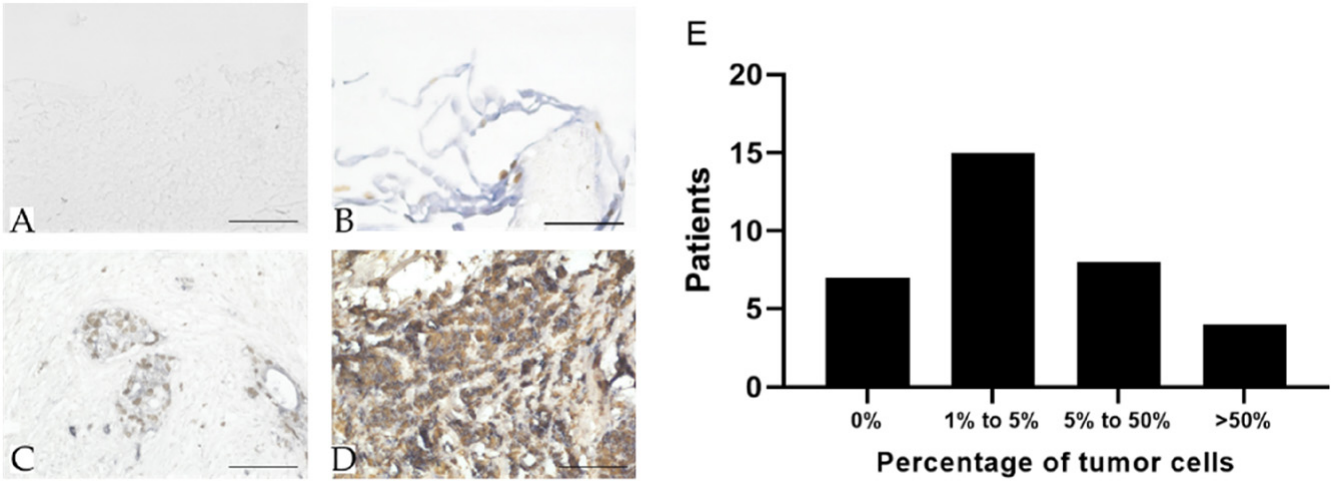
Ki-67-protein and cytokeratin expression in biopsies of GC patients with PM. Tumor cell proportions were classified into four groups: No tumor cells (A), 1–5 % tumor cells (B), 5–50 % tumor cells (C), and >50 % tumor cells (D) (bar – 100 µm). Distribution of the 34 investigated patients in each group (E).

The quantitative variation in these two parameters’ expression patterns was relied upon to perform the efficacy analysis of cytostatic drugs in patients who underwent PIPAC. To evaluate the heterogeneity of the histopathological tumor cell amounts in macroscopic PM lesions (taken during diagnostic laparoscopy), 34 GC patients with PM were documented in a pilot study. Histopathological staining results were classified into four subgroups (Figure 4A–D). Classification was determined by the proportion of tumor tissue to total biopsy material. No tumor tissue was detected in 20.6 % of cases (Figure 4A), whereas we detected a tumor proportion of 1–5 in 44.1 % (Figure 4B); the tumor proportion was 5–50 % in 23.5 % (Figure 4C), and in 11.8 %, the tumor proportion exceeded 50 % (Figure 4D). Absolute numbers are shown in Figure 4E.

### Cisplatin and doxorubicin treatment in patient-derived TSC

**Figure 5:**
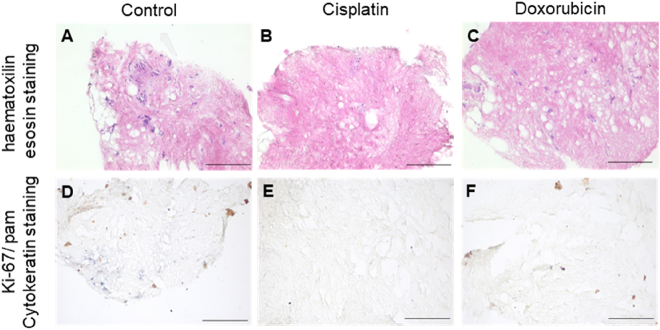
HE and Ki-67/cytokeratin staining in 3D-tissue slice cultures. TSC #57.1: 53-year-old female patient with peritoneal metastatic signet ring cell carcinoma of the stomach, first diagnosed in November 2015, uT2 uN2 cM1 (PER, HEP, OVAR), PCI-score according to Sugarbaker 33 of 39 points and 5,500 mL ascites in the initial laparoscopy. TSCs were treated as control (A/D) and by cisplatin (B/E) and doxorubicin (C/F).

TSCs were treated by cisplatin and doxorubicin for 96 h. Cisplatin and doxorubicin are the standard chemotherapeutic agents used for PIPAC [26]. Cytokeratin and Ki-67 positive cells were analyzed in formalin-fixed specimens. In total, tissue from seven patients was cultured. After exposure to doxorubicin, we detected cytokeratin-positive areas in none of these seven patients. Cisplatin treatment resulted in two cytokeratin positive and five cytokeratin-negative TSCs. Ki-67 expression was present in patients #87 and #89 - in both treatment groups, cisplatin and doxorubicin. We identified no correlation between the *in vitro* chemotherapeutic response rate and overall survival in our cohort. However, patient #59 presented the longest overall survival and was negative for cytokeratin and Ki-67 expression after treatment of TSCs *in vitro* (Table 2). A representative cytokine and Ki-67 staining is given in Figure 5. Cytokeratin and Ki-67 staining in controls are shown in Table 3.

**Table 2:** Histological response of Ki-67 and CK to cisplatin and doxorubicin.

Patient-ID	Overall survival after 1st PIPAC [26]	Cisplatin		Doxorubicin	
		CK	Ki-67	CK	Ki-67
# 57	78 days	neg^a^.	neg.	neg.	neg.
# 59	481 days	neg.	neg.	neg.	neg.
# 65	97 days	pos^b^.	neg.	neg.	neg.
# 81	112 days	neg.	neg.	neg.	neg.
# 87	93 days	pos.	pos.	neg.	pos.
# 89	151 days	neg.	pos.	neg.	pos.
# 90	148 days	neg.	neg.	neg.	neg.

^a^Negative (neg.): cytokeratin and/or Ki-67-positive areas are detectable (no histopathological response). ^b^Positive (pos.): cytokeratin and/or Ki-67-positive not detectable (histopathological response).

**Table 3:** Histopathological response of Ki-67 and CK to oxaliplatin, 5-FU and irinotecan.

Patient-ID	Control		Oxaliplatin		5′Fluorouracil		Irinotecan	
	CK	Ki-67	CK	Ki-67	CK	Ki-67	CK	Ki-67
# 57	pos.	pos.	pos.	pos.	neg.	neg.	neg.	neg.
# 59	neg.	neg.	neg.	neg.	pos.	pos.	neg.	neg.
# 65	neg.	pos.	neg.	neg.	neg.	neg.	neg.	neg.
# 81	neg.	pos.	neg.	neg.	neg.	neg.	neg.	pos.
# 87	pos.	pos.	pos.	pos.	neg.	neg.	pos.	pos.
# 89	neg.	neg.	neg.	pos.	(^a^)	(^a^)	(^a^)	(^a^)
# 90	neg.	pos.	(^a^)	(^a^)	(^a^)	(^a^)	neg.	neg.

^1^Positive (pos.): cytokeratin and/or Ki-67-positive areas detectable (no histopathological response). ^2^Negative (neg.): cytokeratin and/or Ki-67-positive not detectable (histopathological response). ^3^(^a^): no evaluation possible (e.g. insufficient tissue amount).

### TSC treatment with non-standard chemotherapeutic agents

**Figure 6:**
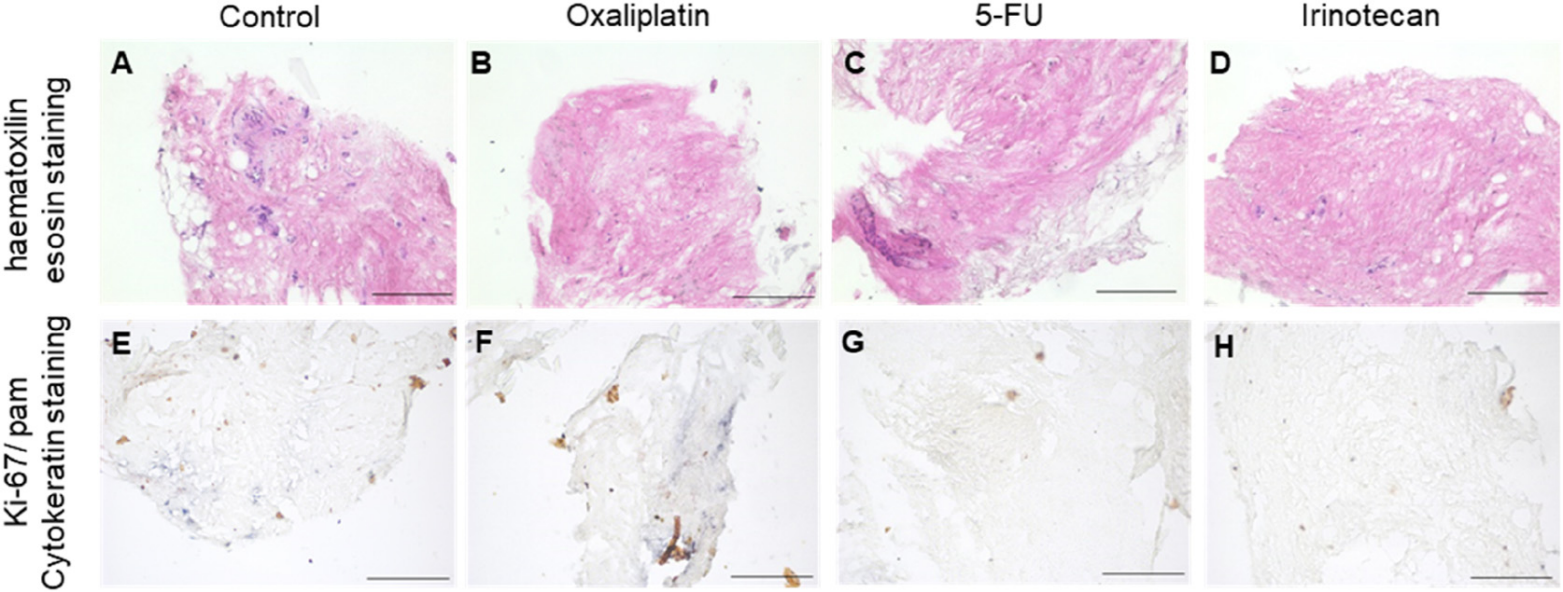
HE and Ki-67/cytokeratin staining in 3D-tissue slice cultures. TSC #57.1: 53-year-old female patient with peritoneal metastatic signet ring cell carcinoma of the stomach, first diagnosed in November 2015, uT2 uN2 cM1 (PER, HEP, OVAR), PCI-score according to Sugarbaker 33 of 39 points and 5,500 mL ascites in the initial laparoscopy. TSCs were treated as control (A/B), by oxaliplatin (B/F), 5-FU (C/G), and irinotecan (D/H). In this case, oxaliplatin treatment failed to reduce numbers of pan-cytokeratin and Ki-67-positive tumor cells, whereas 5-FU and irinotecan treatment reduced both parameters (Ki-67 and pan-cytokeratin).

TSCs were treated with chemotherapeutic agents not employed for PIPAC so far. However, there is some evidence for treating PM patients with colorectal cancer with oxaliplatin during PIPAC. TSCs were treated by 5-FU and irinotecan in addition to oxaliplatin. In oxaliplatin-treated TSCs, a loss of cytokeratin expression was observed in four out of six TSCs, and a loss of Ki-67-expression in three out of six TSCs. 5-FU was proved to lower the cytokeratin and Ki-67 expression in four out of five TSCs. Moreover, irinotecan induced a reduction of the cytokeratin expression in five out of six cases and a reduced number of Ki-67-expressing cells in four of six cases was observed (Table 3, Figure 6).

## Discussion

PM and/or stage IV GC are poor prognostic factors and the overall survival of patients with PM from GC remains poor [27], 28]. More radical onco-surgical techniques and approaches, such as peritonectomy, multivisceral resections, and intraperitoneal chemotherapy, have been attempted in addition to pharmacological interventions [29]. Symptom control and stabilizing the quality of life are the most important clinical goals for these patients. PIPAC is a minimally invasive surgical approach with low operative risk and a short postoperative in-hospital stay with good overall tolerability [25], 26]. Grishally et al. reported that PIPAC might function as preconditioning or in analogy to neoadjuvant therapy before CRS and HIPEC in order to enlarge the indications in the presence of diffuse small-bowel involvement, and that it reduces the extent of tumor-infiltrated tissue to be surgically resected [30]. By administering chemotherapeutics, such as aerosol in a PIPAC setting, the necessary chemotherapeutic drug dose can be reduced to 1:10 of the systemic dose delivered intravenously [31]. Local cytostatic application results in cancer cells being exposed in much closer proximity within the peritoneal cavity, and the systemic concentration can be minimized [31], 32].

In this study, we were able to collect biopsies from GC patients with PM before they underwent their first PIPAC procedure. These biopsies were sliced into TSCs (350 µM), and they were treated by cytostatic drugs, which were used for PIPAC in daily clinical setting (cisplatin and doxorubicin), and to three experimental drugs (5-FU, oxaliplatin and irinotecan) to evaluate the drug response in a patient-derived TSC model.

TSC models display a physiological tumor architecture and heterogeneity, and contain tumor, stroma, and immune cells [33]. TSCs from PM were first evaluated to maintain their morphology in *in vitro* culture for 120 h. Treatment lasted 96 h, after which we evaluated the response to specific chemotherapeutic agents. In our study, we clearly demonstrated that the use of 5-FU, oxaliplatin, and irinotecan triggered an effect resembling that of both cisplatin and doxorubicin, as they are the standard chemotherapeutic compounds used for PIPAC [26]. HE staining is a standard method for pathological examinations. Our histopathologic criteria for malignancy were therefore polymorphia, anisonucleosis, nuclear hyperchromasia, a shift in the nuclear-plasma-relation, and increased basophilia. HE staining is a standard, cheap and quick method for assessing tumor specimens histopathologically. In this experimental approach, HE staining was accompanied by additional immunological staining for pan-cytokeratin and Ki-67. Pan-cytokeratin was used to identify tumor cells of an epithelial origin, and Ki-67 is a biomarker for proliferative active cells [34]. As tumor cells are characterized by uncontrolled cell division, Ki-67 is located to the nucleus and used to identify tumor cells growing during the transition from the G1 to M phase, but not in the G0 phase. Thereby, Ki-67 is associated with tumor development, progression, invasion, metastasis, and prognosis in many cancer entities, such as breast and gastric cancer [35], 36]. There is evidence that Ki-67 upregulation correlates with age, lymph node metastasis, tumor size, the tumor’s TNM stage, and histopathological differentiation in patients with GC [37]. However, Ki-67 is a potentially heterogeneous prognostic marker depending on different cut-off values, sample size, and whether whole slides or tissue micro arrays have been used [35]. In our study, when it was present, we detected strong Ki-67 expression in PMs from GC patients. Signet ring cell GC is often detected in conjunction with a PM, and patients suffer from CDH1 mutations, which encode for E-cadherin, an adhesion protein responsible for cell-cell contact [29]. When downregulated, E-cadherin itself is also associated with metastatic processes, as the loss of the cell-cell contact enables tumor cells to escape from their tumor to undergo EMT (epithelial–mesenchymal transition) [38]. Since the introduction of immunotherapies, investigations of these cell types have increased. TSCs are an ideal model to study the complex interaction between tumor and immune cells [19], 23], 39].

Human patient-derived *ex vivo* models for PM are rare [40], [41], [42], [43], [44], because an invasive approach (surgery) is necessary to obtain PM tissue, and obtaining a re-biopsy from the same tumor area is usually impossible. Other methods, such as endoscopy, can be applied to obtain tumor tissue from gastrointestinal cancers. Retrieving ascites fluid can facilitate the isolation of tumor cells from PMs. However, this contains tumor cells only, which have abandoned their tumor organization, thus making them a sub-group of PM tissue. A co-culture system of peritoneal surface and patient-derived colorectal cancer organoids has recently been shown, namely that HIPEC conditions (41 °C) increased the cellular intake of doxorubicin and a decrease in invading cells [44]. The advantage of TSCs is that they are robust, can be easily processed and analyzed in a few days, and are not cost-intensive. Other models, such as organoids, are dependent on highly specific expert-knowledge, while they are labor-, time-, and cost-intensive.

The strengths of our study include the availability of PM tissue to set up a human *ex vivo* model for PM biopsies from patients with GC. In this setting, we were able to evaluate the chemotherapeutic drug response. Nevertheless, we believe that this model will prove to be suitable for investigating PMs from other cancer entities too, such as colorectal, pancreatic, and ovarian cancer.

## Conclusions

In summary, we clearly demonstrated that TSCs from PM from patients with GC are a suitable model from which to assess the drug response. TSCs from PMs can be applied under various culture conditions, and the drug response can be visualized applying robust methods like HE-staining and standard immunohistochemistry with pan-cytokeratin and Ki-67. Nevertheless, more profound insight and sophisticated research is needed to predict chemotherapeutic response rates in patients with PMs from GC and other solid cancers.
